# MistGo^®^ Compared to Conventional Eye Drops: A Patient-Reported Evaluation of Comfort and User-Friendliness in Glaucoma Treatment

**DOI:** 10.3390/jcm15010067

**Published:** 2025-12-22

**Authors:** Astrid Dissing Sjö, Marie Louise Holm Møller, Rune Nørager, Nicolai Sjö, Miriam Kolko

**Affiliations:** 1 Department of Drug Design and Pharmacology, University of Copenhagen, 2100 Copenhagen, Denmark; astriddissingsjo@gmail.com; 2 Design Psychology APS, 1801 Frederiksberg, Denmark; marielouise@designpsykologi.dk (M.L.H.M.); rune@designpsykologi.dk (R.N.); 3 Øjenlæge Nicolai Sjö, 1107 Copenhagen, Denmark; nicolai.sjo@gmail.com; 4 Your Eye Doctors, Eye Hospital Rungsted Kyst, 2960 Rungsted Kyst, Denmark; 5 Department of Ophthalmology Aalborg University Hospital, 9000 Aalborg, Denmark; 6 Department of Ophthalmology, Rigshospitalet, 2600 Glostrup, Denmark

**Keywords:** ocular hypertension, glaucoma, adherence, drug delivery device, comfort, user-friendliness, eye drops

## Abstract

**Background/Objectives**: Poor adherence remains a key challenge in glaucoma management, often due to difficulties with accurate self-administration and discomfort associated with conventional eye drop bottles. MistGo^®^ is a novel dispensing device that delivers precise micro-doses of medication as a fine mist, allowing dosing with a neutral head position. With its ergonomic design, eye rest, and dose release button, MistGo^®^ aims to improve comfort and ease of use. This study compared the user-friendliness, comfort, and administration confidence of MistGo^®^ versus conventional eye drop dispensers in patients using topical ocular hypotensive medications. **Methods**: Twenty-two patients with glaucoma or ocular hypertension who had used conventional eye drop dispensers for ≥3 months were enrolled. Participants used the MistGo^®^ device for 14 days and subsequently rated comfort, user-friendliness, and administration confidence on 0–10 scales for both their conventional dispensers and the MistGo^®^ device. **Results**: MistGo^®^ was rated significantly higher than conventional eye drop dispensers in terms of comfort (*p* < 0.0001), caused less discomfort from excess fluid (*p* < 0.001), and was perceived as more user-friendly (*p* < 0.001). There was no significant difference in the perceived accuracy of administration (*p* = 0.5); however, participants reported a significantly lower likelihood of medication being applied outside the eye when using MistGo^®^ (*p* < 0.001). Overall, 20 out of 22 patients preferred MistGo^®^. **Conclusions**: Patients with glaucoma or ocular hypertension preferred MistGo^®^ over conventional eye drop dispensers as they found it more comfortable and user-friendly. These findings suggest that MistGo^®^ has the potential to reduce barriers to adherence in glaucoma care. Further studies are warranted to evaluate its long-term efficacy and broader applicability.

## 1. Introduction

Glaucoma is the leading cause of irreversible vision loss worldwide, affecting millions of people, particularly older adults [1,2]. While there is no cure, disease progression can be slowed through lifelong use of topical ocular hypotensive medications, such as prostaglandin analogues and beta blockers, often administrated multiple times daily [1,2]. These medications are also commonly prescribed to patients with ocular hypertension (OHT), a condition characterized by elevated intraocular pressure without optic nerve damage, to reduce the risk of developing glaucoma [1,3]. Despite the proven efficacy of these medications in lowering intraocular pressure (IOP) and slowing disease progression, adherence to prescribed treatment regimens remains a major challenge [4,5]. Although consistent daily use is critical, a study found that only 56% of patients administered more than 75% of their prescribed doses [6], while other studies report deviations from prescribed regimens ranging from 5% to 80%, depending on the method used to quantify adherence [7,8,9,10].

One significant barrier to adherence is the difficulty many patients experience when using conventional eye drop bottles [4,11,12]. Although findings vary across studies, some report that only about 30% of patients properly instil their medication as prescribed [12]. Many struggles with proper administration, such as missing the eye altogether or inadvertently contaminating the bottle tip by allowing it to come into contact with the eye [4,11,12,13,14,15]. These challenges are particularly pronounced among elderly patients, who often struggle with conditions such as arthritis and limited neck mobility, further complicating the administration process [4,15]. A study found that 17% of the patients using ocular hypotensive eye drops relied on help from others for administration [13]. Additionally, the need for daily administration, along with local side effects such as hyperemia, eye irritation, and skin irritation due to excessive liquid overflow can make adherence challenging [16]. As a result, many patients fail to achieve optimal IOP control, increasing the risk of vision loss [4]. Furthermore, systemic absorption through the nasolacrimal duct, which is partly caused by the large drop size and excessive medication overflow, allows the drug to enter the bloodstream directly via the mucosa, bypassing hepatic first-pass metabolism. This can increase the risk of serious systemic side effects, such as bradycardia and orthostatic hypotension, ultimately compromising patient safety [17,18,19].

Given these challenges, there is a pressing need for innovative drug delivery systems that enhance patient compliance and treatment effectiveness. MistGo^®^ is an innovative alternative to conventional eye drop dispensers, designed to improve the precision and ease of administration regardless of head position. The device incorporates usability features such as a slide-button release mechanism and an integrated eye rest for correct positioning, eliminating the need for head tilting and reducing hand–eye coordination demands. The integrated eye rest provides a fixed point of contact at the orbital rim, thereby standardizing the distance and angle between the nozzle and the ocular surface. This ensures proper alignment across users and minimizes common dosing errors associated with conventional bottles. For example, it prevents inadvertent contact between the tip and the eye or periocular skin, which can increase the risk of contamination, and minimizes the likelihood of misalignment of the dispenser that can cause drops to miss the ocular surface. Mist dispersion is standardized through a micro-engineered nozzle that generates a reproducible aerosol cone, enabling consistent droplet size distribution and uniform corneal coverage independent of head orientation.

Conventional eye drop bottles and ampules dispense 25–70 μL per dose [19,20]. However, studies on microdosing have demonstrated that much smaller volumes (5–15 μL) can achieve therapeutic effects comparable to conventional eye drops volumes [17,18,21,22,23,24]. MistGo^®^ delivers a consistent 6-μL dose of medication in a fine mist, promoting even corneal coverage while minimizing excess fluid runoff.

Microdosing optimizes drug utilization while reducing medical waste, systemic absorption, cost of the therapy, and adverse events [18,19,23], as both the incidence and severity of local and systemic side effects correlate with drop size [18].

These aspects may help address key barriers to proper instillation and improve treatment adherence while reducing the risk of adverse events, both systemically and locally, making treatment safer. Additionally, MistGo^®^ is compatible with both preservative-free and preserved medications while maintaining sterility, allowing for broader patient use.

To assess patients’ experience using MistGo^®^ compared to conventional eye drop dispensers, we conducted a comparative user experience study. By addressing limitations of conventional eye drop administration, MistGo^®^ may enhance patient compliance, potentially contributing to better disease management, reduced disease progression, and an improved quality of life for patients with glaucoma or ocular hypertension. This study aims to evaluate how MistGo^®^ performs compared to conventional eye drop dispensers with respect to user-friendliness, comfort, and confidence in administration.

## 2. Materials and Methods

### 2.1. Ethics

The study was conducted in accordance with current laws and applicable standards, including the ethical principles originating from the Declaration of Helsinki (1964), last amended at the 64th WMA General Assembly in Fortaleza, October 2013. The study also complied with EU MDR 2017/745 [25] and ISO 14155:2020 [26], which outlines good clinical practices for clinical investigations of medical devices involving human subjects. The study was assessed by the Danish Medicines Agency and the Ethical Committee and deemed non-notifiable.

### 2.2. Participants

A total of 22 patients diagnosed with glaucoma or ocular hypertension (OHT) and in monotherapy with a prostaglandin analogue were recruited from an ophthalmologist in Copenhagen, Denmark. Eligible participants were required to be at least 18 years of age, possess full legal capacity, and be Danish speaking. Additionally, they must have been receiving a prostaglandin analogue as topical ocular hypotensive treatment for a minimum of three months prior to enrollment, either in multi-dose bottles or single-use containers. Therefore, the term “eye drop dispensers” in the following sections refers to a mix of both formats.

Exclusion criteria included pregnancy or breastfeeding, the presence of other ocular diseases, ocular surgery within the preceding three months, or dermatological conditions affecting the periocular area. To minimize selection bias, the ophthalmologist consecutively screened clinic patients who met the inclusion criteria, without prior knowledge of their satisfaction with eye drop treatment. Eligible patients were invited to participate and provided both oral and written informed consent prior to enrollment.

### 2.3. Study Design and Data Collection

This clinical study aimed to evaluate the patients’ experience of using MistGo^®^ (EYE-GO; Hoersholm, Denmark). Over a 14-day period, participants replaced their usual eye drop bottles with MistGo^®^ containing Tafluprost (Taflotan^®^ Sine; Santen Oy, Tampere, Finland), a prostaglandin analogue. The participants were instructed on how to use MistGo^®^ correctly and were told to administer the medication in one or both eyes according to their usual regimen. Although individual instructions allowed for either option, all participants habitually treated both eyes and therefore used MistGo^®^ bilaterally throughout the study. They were also asked to record the time of administration each day. After the study period, the participants rated both MistGo^®^ and their usual eye drop dispensers on various parameters including comfort, user-friendliness, and confidence in administration using a numerical scale from 0 to 10. Furthermore, the participants were asked to report their overall preference between MistGo^®^ and their usual eye drop dispenser. All interviews were administered by an investigator affiliated with EYE-GO. A standardized neutral script was used, and only predefined quantitative rating questions were asked. Participants rated their conventional eye drop dispenser and MistGo^®^ side by side on the same 0–10 scale for each question.

### 2.4. Equipment

The primary study material was MistGo^®^, a mechanical eye dispenser developed by EYE-GO and designed for micro-dose administration of ophthalmic medication (precisely 6 µL per use). In this study, MistGo^®^ was filled with Tafluprost, a prostaglandin analogue that lowers the intraocular pressure by increasing aqueous fluid outflow.

**Figure 1:**
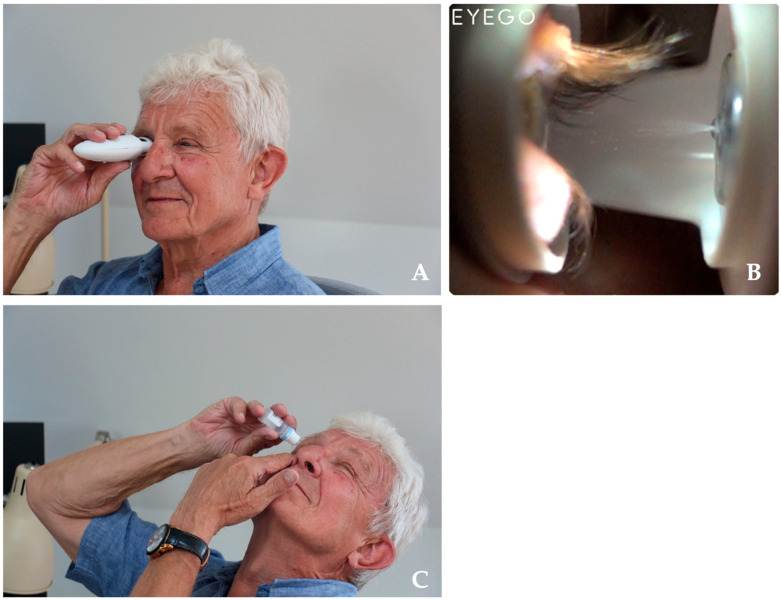
(**A**) Demonstration of MistGo^®^ usage. The thumb is used to slide the button that releases the dose. (**B**) Close-up of mist application with MistGo^®^ onto the ocular surface. (**C**) Demonstration of conventional eye drop bottle usage, where one hand pulls down the lower eye lid while the other hand squeezes the bottle. Written consent was obtained for use of photos.

MistGo^®^ is administered by loading a dose, positioning the guiding eyepiece around the eye, and sliding the button to release the mist into the eye (Figure 1A). The dose is limited to what the eye can absorb and is delivered before the blink reflex is triggered (Figure 1B). MistGo^®^ can be used in a natural head position, eliminating the need to tilt the head back [27]. In contrast, conventional eye drop dispensers require the patient to tilt the head back, pull down the lower eyelid with one hand, and use the other hand to squeeze a drop from the bottle into the eye (Figure 1C). After instillation, the eye should remain closed, while using a finger to press on the nasolacrimal duct to reduce the risk of systemic absorption [28].

### 2.5. Statistical Analysis

Statistical analyses were performed in R version 4.5.2 (The R Foundation for Statistical Computing; Vienna, Austria) [29]. A paired Wilcoxon signed-rank test (two-tailed) was applied to compare the differences between MistGo^®^ and conventional eye drops for all five endpoints: comfort, no discomfort due to excess fluid, user-friendliness, administration confidence, and no medication hitting outside the eye. The difference between MistGo^®^ and conventional eye drops was considered significant if *p* < 0.05.

To complement the statistical test, the effect size for each endpoint was estimated using a matched-pair Rank-Biserial Correlation (r_rb_) calculated using the TOSTER package (version 0.8.6) in R 4.5.2 [30,31]. For r_rb_, values range from −1 to +1, indicating how consistently one dispenser outperforms the other, with 0 meaning no difference, and −1/+1 meaning complete dominance of one device over the other (i.e., all participants rated a specific higher than the other). Medians and interquartile ranges (IQR) are reported for all endpoints.

A subgroup analysis was conducted to examine whether participants’ prior experience with dispenser type (multi-dose bottle vs. single-use dispenser) influenced their perceptions of MistGo^®^. Group differences were assessed using the Wilcoxon rank-sum test, and the Rank-Biserial Correlation was calculated as a measure of effect size. The results are reported in Supplementary Table S1. For each subgroup, medians and IQRs are also reported.

An a priori sample size calculation was performed using G*Power 3.1 [32]. For a paired, two-tailed Wilcoxon signed-rank test, we based our assumptions on prior literature, wherein authors also compared the ease of use between a microdroplet device and a conventional eyedropper on a 0–10 scale [33], with an expected mean difference of 2.9 points, and a conservative standard deviation of 2.9. With an alpha level of 0.05 and desired power exceeding 95%, the calculation indicated that 16 participants would be required. In this study, 22 participants were included, resulting in a power exceeding 97%.

## 3. Results

### 3.1. Characteristics of the Study Population

A total of 22 subjects were enrolled in the study, comprising 15 females and 7 males. Ages ranged from 39 to 88 years, with a mean age of 69 years (SD = 10.2). All patients were undergoing treatment with a prostaglandin analogue prior to enrollment, either Taflotan, Monoprost, Xalatan, Latanoprost “Stada” or Lumigan. Five of the participants used a multi-dose bottle as medication dispenser, while the remaining 17 participants used single-use containers. All participants administered MistGo^®^ once daily as instructed. Detailed participant characteristics are presented in Table 1.

### 3.2. Evaluation of Subjects’ Perception of Comfort

The participants’ perception of comfort when using MistGo^®^ to administer Taflotan^®^ as a mist versus as a drop from the conventional eye drop dispensers was rated on a scale from 0 (very uncomfortable) to 10 (very comfortable). The mist from MistGo^®^ was rated significantly more comfortable than the drop from conventional eye drop dispensers (V = 190, *p* < 0.0001, 95% CI [2.0, 3.5], r_rb_ = 0.98). MistGo^®^ received a median rating of 10 (IQR = 1), which was 43% higher than the median rating of 7 (IQR = 1.8) for the conventional eye drop dispensers.

Furthermore, the large effect size (r_rb_ = 0.98) shows that MistGo^®^ was consistently rated more comfortable than conventional eye drops dispensers by almost all participants, with only a few rating them equally comfortable.

Additionally, participants reported significantly less discomfort due to excess fluid when using MistGo^®^ compared to conventional eye drop dispensers (V = 190, *p* < 0.001, 95% CI [3.5, 6.0], r_rb_ = 0.98). On average, MistGo^®^ was rated 67% better than conventional eye drop dispensers on this aspect, since MistGo^®^ received a median rating of 10 (IQR = 0) on the scale from 0 (very bothered by excess fluid) to 10 (not bothered at all), while the median rating of conventional eye drop dispensers was 6 (IQR = 3.8). As reflected by the large effect size (r_rb_ = 0.98), participants were consistently less bothered by excess fluid when using MistGo^®^, with only a small number reporting no difference in levels of discomfort due to excess fluid between MistGo^®^ and conventional eye drops.

### 3.3. Evaluation of Subjects’ Perception of User Friendliness

User-friendliness was assessed on a scale from 0 (not user-friendly at all) to 10 (very user-friendly). On average, the participants rated MistGo^®^ as significantly more user-friendly than conventional eye drop dispensers (V = 169, *p* < 0.001, 95% CI [2.0, 5.0], r_rb_ = 0.91). MistGo^®^ received a median rating of 10 (IQR = 1), which is 43% higher than the median rating of 7 (IQR = 3.8) for the conventional eye drop dispensers. The large effect size (r_rb_ = 0.91) reflects that most participants rated MistGo^®^ as more user-friendly than conventional eye-drop dispensers, with a few rating them equally and only one preferring the conventional eye drop dispenser.

### 3.4. Evaluation of Subjects’ Perception of Administration Confidence

To evaluate participants’ confidence in administering the medication correctly, they were asked how certain they felt that the medication was correctly administered to the eye using MistGo^®^ compared to conventional eye drop dispensers on a scale from 0 (not certain at all) to 10 (very certain). There was no significant difference in perceived accuracy of administration between the two devices (V = 81, *p* = 0.5, 95% CI [−1.0, 2.0], r_rb_ = 0.1). Participants reported high confidence with both devices, and both MistGo^®^ and conventional eye drop dispensers received a median score of 9 (IQR = 2).

**Figure 2:**
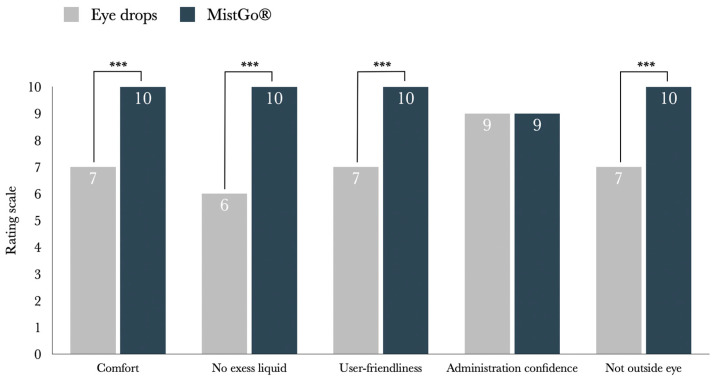
Median ratings of MistGo^®^ versus conventional Eye Drops on a scale from 0 to 10, where 10 represents the best score. Statistically significant differences at *p* < 0.001 are marked with ***.

Participants were also asked to rate the extent to which they experienced medication landing outside the eye, using a scale from 0 (to a very large extent) to 10 (not at all). MistGo^®^ received a median score of 10 (IQR = 1), while conventional eye drop dispensers received a lower median score of 7 (IQR = 3.8). This difference was statistically significant (V = 171, *p* < 0.001, 95% CI [2.0, 4.5], r_rb_ = 0.96) and reflected a large effect size, as all participants rated MistGo^®^ either equal to or better than conventional eye drop dispensers. The results are illustrated in Figure 2.

In addition, a subgroup analysis stratified by prior dispenser type revealed no significant differences in ratings between participants who had previously used multi-dose bottles and those who had used single-use containers (Supplementary Table S1).

## 4. Discussion

This study evaluated the perceived comfort, user-friendliness, and administration confidence when delivering topical ocular hypotensive drugs using the MistGo^®^ micro-dose dispenser, compared to using conventional eye drop dispensers.

We found that MistGo^®^ was perceived as significantly better than conventional eye drop dispensers in terms of comfort and user-friendliness. Participants also reported reduced discomfort from excess fluid runoff and fewer instances of medication missing the eye with MistGo^®^. Overall, 20 out of 22 participants preferred MistGo^®^ over their usual eye drop dispenser.

These results highlight MistGo^®^’s potential to improve patient experience and address common issues associated with conventional eye drop administration, such as poor targeting, excess fluid runoff, and handling difficulties [4,11,12,13,14].

Other devices are available to reduce drop volume and better match the eye’s absorptive capacity, with Nanodropper (Nanodropper, Inc; Rochester, MN, USA) being an FDA-listed cap that attaches to conventional eye-drop bottles [34,35]. MistGo^®^ shares this goal but delivers a consistent 6-μL mist with an integrated eye rest, standardizing alignment and eliminating the need for head tilt. Unlike Nanodropper, which still depends on user positioning, bottle technique, and carries a risk of tip contact and contamination, MistGo^®^ provides reproducible delivery regardless of head position. These features suggest that MistGo^®^ may address gaps in adaptor-based microdosing systems, particularly for patients with limited dexterity or difficulty administering conventional drops.

Improved user-friendliness is especially relevant in glaucoma care, where difficulties with eye drop administration is a well-known barrier to consistent use of glaucoma medications [4,14,15], potentially leading to suboptimal IOP control and increased risk of disease progression. By eliminating the need to tilt the head back and aim precisely, MistGo^®^ may support better long-term adherence, particularly among elderly patients or those with visual, motor, or coordination difficulties. Additionally, improved user-friendliness may help expand access to topical eye medications in contexts where self-administration is challenging, such as in pediatric care or post-surgical patients, where user-friendliness and hygiene are critical.

Interestingly, although participants reported significantly higher comfort, user-friendliness, and accuracy with MistGo^®^, there was no significant difference in administration confidence between MistGo^®^ and conventional eye drop dispensers. Both MistGo^®^ and conventional eye drop dispensers received a median score of 9 (IQR = 2). Several factors may help explain this finding. One possibility is familiarity bias, namely the tendency to favour or feel more confident in a method that is well-known or long-practiced [36]. Participants had been using conventional eye drop dispensers for at least three months prior to the study, whereas MistGo^®^ was used for only 14 days, which may contribute to the persistently high confidence ratings for the familiar device. This interpretation is consistent with prior research showing that many patients do not administer eye drops correctly despite reporting high confidence [6].

At the same time, an alternative explanation is the stronger sensory feedback produced by conventional drops. The larger liquid volume provides a clear tactile cue that many patients associate with successful dosing, which may reinforce confidence irrespective of actual technique. Notably, despite delivering a smaller, less perceptible mist and being entirely unfamiliar at baseline, MistGo^®^ still achieved a very high confidence rating after only a short trial period. This suggests that users were able to rapidly develop trust in the device even without the strong sensory cues provided by conventional drops.

This study focused on patient-reported outcomes, such as user-friendliness, comfort, and preference, which are inherently subjective. We considered it relevant to first evaluate whether patients favoured the design and ergonomics of the micro-dosing device before undertaking larger-scale studies involving objective clinical measures such as intraocular pressure (IOP), tear-film stability, blink dynamics, or dosing accuracy. While these objective outcomes were beyond the scope of the current study, future research should incorporate them to complement patient-reported measures and provide a more comprehensive evaluation of the device’s performance. While most studies on micro-dosing delivery systems have primarily focused on clinical outcomes such as IOP reduction and decreased systemic absorption [17,18,21,31], there is some evidence pointing to improved patient experience as well. For example, Ianchulev et al. (2016) reported that patients using a micro-dose dispenser experienced greater comfort related to head positioning and less tearing or overflow during administration [33]. Although limited, these findings align with our results and support the notion that micro-dosing devices like MistGo^®^ may enhance patient comfort and user-friendliness compared to conventional eye drop dispensers [4,14,15].

### Limitations

Nonetheless, limitations to our study should be acknowledged. First, there is a substantial potential for interviewer bias, as the interviewer was employed by EYE-GO, the company that developed MistGo^®^. This affiliation may have unintentionally influenced participant responses, particularly for subjective outcomes such as comfort, preference, and user-friendliness. Although interviews followed a standardized set of quantitative questions and responses were analyzed by an independent third party, the possibility of inadvertent interviewer influence cannot be excluded. Future studies should employ independent, blinded assessors to minimize this risk.

Second, participants were using different types of prostaglandin analogues prior to the study (e.g., Taflotan, Lumigan, Monoprost), which may differ in side effect profiles and could have influenced baseline perceptions of comfort. Similarly, participants were using different types of dispensers prior to the study (5 participants used a multi-dose bottle while the remaining 17 participants used single-use containers). These dispenser formats differ in several aspects of user experience, including opening mechanism, ease of positioning and aiming, and force required to dispense a drop. A stratification of participants into subgroups (multi-dose vs. single-use) revealed no significant effect of previous device on their experience with MistGo^®^ (see Table S1). Given the small number of participants in the multi-dose subgroup (n = 5), these analyses should be interpreted with caution and are considered exploratory. Future studies with larger subgroup sizes are needed to provide greater statistical power to explore potential subgroup differences more thoroughly.

Third, the study was conducted with a relatively small sample of 22 participants, which limits the generalizability of the findings. A larger sample could provide more robust insights into the user satisfaction with MistGo^®^ across different patient demographics. However, it is worth noting that several key outcomes in this study demonstrated highly significant differences (e.g., *p*-values < 0.01), along with large effect sizes, suggesting that the observed effects were robust even within this limited sample. Additionally, the participants were highly representative of the intended user population in terms of age. Participants’ ages ranged from 39 to 88 years, with a mean age of 69 years (SD = 10.2), capturing both middle-aged and older adults rather than exclusively including older patients with reduced mobility. This diversity supports the generalizability of the observed effects despite the modest sample size.

In addition, participants consistently rated MistGo^®^ high on user-friendliness (median = 10, IQR = 1), while there was a higher disagreement between participants when it came to the user-friendliness of conventional eye drop dispensers (median = 7, IQR = 3.8). The very small interquartile range for MistGo^®^ indicates that all participants found MistGo^®^ consistently user-friendly, with ratings ranging from 7 to 10. In contrast, the larger variability in ratings for conventional eye drop dispensers (ranging from 2 to 10) reflects more diverse user experiences, indicating that while some participants had no difficulties, others found them significantly challenging to use. This shows that the study included both patients who were comfortable with their existing eye drop regimen and patients who were not. However, we did not collect detailed baseline information on participants’ underlying ability to instil drops (e.g., manual dexterity, tremor, visual acuity, or prior experience). These factors may influence perceived ease of use and should be evaluated in future studies to help identify which patient subgroups may benefit most. All participants rated MistGo^®^ either higher or equal to their conventional eye drop dispenser, suggesting that its advantages were recognized regardless of prior satisfaction with conventional methods, and that its acceptability is not limited to those who were dissatisfied with their usual eye drop dispenser.

Fourth, the participants were instructed on how to use the MistGo^®^ device, which may have influenced their perceptions and ease of use. In real-world settings, users who might not receive demonstration of how to use the device may experience different outcomes. Finally, the short duration of the study captures only early user experience and limits our understanding of the long-term effects of using MistGo^®^. A longer study could offer valuable insights into whether MistGo^®^’s benefits are sustained over time and if they translate into improved IOP control and patient adherence in the long run.

Despite these limitations, this study offers encouraging evidence that MistGo^®^ represents a comfortable, user-friendly, and innovative alternative to conventional eye drop dispensers. Microdosing has demonstrated therapeutic effects comparable to conventional eye drops volumes [17,18,21,22,23,24]; however, future research should further evaluate the clinical efficacy of microdosing ocular eye drops with MistGo^®^, particularly its ability to provide sustained IOP reduction in patients with glaucoma. Beyond glaucoma, MistGo^®^ also holds strong potential for the delivery of other eye drops, including lubricants for dry eye disease and postoperative antibiotics. Although the current MistGo^®^ model delivers a fixed 6-μL dose, the underlying mechanism is volume-scalable, and minor design modifications could enable future versions to dispense alternative microdose volumes (e.g., 8–10 μL) if required for specific treatments.

## 5. Conclusions

This study demonstrates that MistGo^®^ offers significant improvements in both comfort and user-friendliness compared to conventional eye drop dispensers for patients with glaucoma or OHT. Overall, 20 out of 22 patients preferred MistGo^®^ over their usual eye drop dispenser. After 14 days of using MistGo^®^, patients reported significantly higher ratings of comfort and user-friendliness with MistGo^®^, and significantly less discomfort from excess fluid and fewer cases of getting medication outside the eye.

Despite the promising results, limitations such as the short study duration and potential interviewer bias should be acknowledged. Future studies using blinded assessors with extended follow-up periods and measurements of the intraocular pressure would be valuable in assessing the long-term safety and efficacy of administration with MistGo^®^. Moreover, exploring its use with other ocular medications, such as lubricants for dry eye disease, could further expand MistGo^®^’s potential applications.

Overall, MistGo^®^ represents a promising alternative to conventional eye drop administration by making ocular medication more comfortable and easier to use. A 6-month, assessor-blinded, randomized controlled trial comparing IOP control and electronically monitored adherence between MistGo^®^ and standard care is warranted to assess its long-term efficacy, evaluate its role in reducing barriers to adherence, and determine its impact on overall glaucoma treatment outcomes.

## Figures and Tables

**Table 1:** Baseline patient characteristics.

	Participants, n = 22
Age	
Mean (SD)	69 (10.2)
Sex	
Female, n (%)	15 (68%)
Male, n (%)	7 (32%)
Eye disease	
Ocular hypertension, n (%)	10 (45%)
Glaucoma, n (%)	12 (55%)
Prostaglandin analogue used before enrollment	
Taflotan, n (%)	9 (41%)
Monoprost, n (%)	10 (41%)
Latanoprost “Stada”, n (%)	1 (4.5%)
Xalatan, n (%)	1 (4.5%)
Lumigan, n (%)	1 (4.5%)
Eye drop dispenser used before enrollment	
Single-use container, n (%)	17 (77%)
Multi-dose bottle, n (%)	5 (23%)

## Data Availability

The original contributions presented in this study are included in the Supplementary Material. Further inquiries can be directed to the corresponding authors.

## Supplementary Materials

The following supporting information can be downloaded at: https://www.mdpi.com/article/10.3390/jcm15010067/s1, Table S1: Sub-group analysis based on participants’ previous dispenser type.

## Abbreviations

The following abbreviations are used in this manuscript (in alphabetic order):

CI	Confidence Interval
IOP	Intraocular pressure
IQR	Interquartile range
M	Median
OHT	Ocular Hypertension
r_rb_	Rank-Biserial Correlation
SD	Standard deviation
