# Supplementary material for: MistGo® Compared to Conventional Eye Drops: A Patient-Reported Evaluation of Comfort and User-Friendliness in Glaucoma Treatment
Source: J Clin Med. 2025 Dec 22;15(1):67. doi: 10.3390/jcm15010067 (PMC12786802; doi:10.3390/jcm15010067)
Supplement: Supplementary file 1 [file jcm-15-00067-s001.zip › jcm-4049003-supplementary.pdf]

Table S1: Sub-group analysis based on participants' previous dispenser type. Median (interquartile range) are shown for eye drops and MistGo® for both the multi-dose and single-use group. A Wilcoxon rank sum test (equivalent to the Mann-Whitney test) and a Rank-Biserial Correlation (r_{rb}) are used to investigate whether the type of previous dispenser affected how participants rated MistGo®. With only 5 participants in the multi-dose group, power is very limited, and these comparisons are therefore considered exploratory.

	**Multi-dose group (N=5)**		**Single-use group (N=17)**		**Comparison:** Did previous dispenser affect rating of MistGo®?
	Eyedrops	MistGo	Eyedrops	MistGo	
Comfort	8 (1)	9 (1)	7 (2)	10 (1)	$p = 0.3$, $r_{rb} = -0.27$
No excess liquid	7 (6)	10 (0)	5 (3)	10 (0)	$p = 0.5$, $r_{rb} = 0.11$
User Friendliness	6 (4)	10 (2)	7 (3)	10 (1)	$p = 0.7$, $r_{rb} = -0.11$
Administration confidence	8 (2)	9 (2)	10 (2)	9 (2)	$p = 1$, $r_{rb} = 0.01$
Not outside eye	7 (4)	10 (1)	7 (3)	10 (1)	$p = 0.8$, $r_{rb} = 0.07$
